# Pericardial tamponade, a diagnostic chameleon: from the historical perspectives to contemporary management

**DOI:** 10.1186/s13019-023-02174-9

**Published:** 2023-02-04

**Authors:** Ann-Sophie Kaemmerer, Khaleel Alkhalaileh, Mathieu N. Suleiman, Markus Kopp, Christine Hauer, Matthias S. May, Michael Uder, Michael Weyand, Frank Harig

**Affiliations:** 1 Department of Cardiac Surgery, University Hospital Erlangen, Friedrich-Alexander-University Erlangen-Nürnberg, Krankenhausstrasse 12, 91054 Erlangen, Germany; 2 Institute of Radiology, University Hospital Erlangen, Friedrich-Alexander-University Erlangen-Nürnberg, Erlangen, Germany

**Keywords:** Cardiac tamponade, Postoperative complication, Pericardial effusion, Diagnostics, Historical aspects

## Abstract

**Background:**

Pericardial tamponade (PT) early after cardiac surgery is a challenging clinical entity, not infrequently misrecognized and often only detected late in its course. Because the clinical signs of pericardial tamponade can be very unspecific, a high degree of initial suspicion is required to establish the diagnosis. In addition to clinical examination the deployment of imaging techniques is almost always mandatory in order to avoid delays in diagnosis and to initiate any necessary interventions, such as pericardiocentesis or direct cardiac surgical interventions. After a brief overview of how knowledge of PT has developed throughout history, we report on an atypical life-threatening cardiac tamponade after cardiac surgery. A 74-year-old woman was admitted for elective biological aortic valve replacement and aorto-coronary-bypass grafting (left internal mammary artery to left anterior descending artery, single vein graft to right coronary artery). On the 10th postoperative day, the patient unexpectedly deteriorated. She rapidly developed epigastric pain radiating to the left upper abdomen, and features of low peripheral perfusion and shock. There were no clear signs of pericardial tamponade either clinically or echocardiographically. Therefore, for further differential diagnosis, a contrast-enhanced computed tomography scan was performed under clinical suspicion of acute abdomen. Unexpectedly, active bleeding distally from the right coronary anastomosis was revealed. While the patient was prepared for operative revision, she needed cardiopulmonary resuscitation, which was successful. Intraoperatively, the source of bleeding was located and surgically relieved. The subsequent postoperative course was uneventful.

**Conclusions:**

In the first days after cardiac surgery, the occurrence of life-threatening situations, such as cardiac tamponade, must be expected. Especially if the symptoms are atypical, the entire diagnostic armamentarium must be applied to identify the origin of the complaints, which may be cardiac, but also non-cardiac.

**Central message:**

A high level of suspicion, immediate diagnostic confirmation, and rapid treatment are required to recognize and successfully treat such an emergency (Fig. 5).

**Perspective:**

Pericardial tamponade should always be considered as a complication of cardiac surgery, even when symptoms are atypical. The full range of diagnostic tools must be used to identify the origin of the complaints, which may be cardiac, but also non-cardiac (Fig. 5).

## Introduction

Early postoperative pericardial tamponade is a life-threatening emergency of paramount importance to any cardiac surgeon dealing caring for either acquired or congenital heart disease. Cardiac tamponade, defined by a fluid accumulation in the pericardial sac, is requiring early diagnosis and immediate action. This condition has multiple causes, including postoperative bleeding after cardiac surgery. Cardiac tamponade produces an increase of the intrapericardial pressure, a compression of the heart with cardiac inflow restriction, eventually causing organ failure, shock, and cardiac arrest in extreme cases. In pericardial tamponade, an accurate and rapid diagnosis is critical, but it may take some time before noticeable signs and symptoms develop, especially if the fluid is slowly increasing. Unfortunately, the results of the physical examination are not very conclusive, even in the presence of major pericardial effusion or tamponade [1].

## Case presentation

A 74-year-old obese female patient (160 cm, 91,4 kg, BMI 36 kg/m^2^) was admitted for planned coronary bypass grafting (CABG) and aortic valve replacement. Communication was very limited because of a lack of linguistic abilities. The preoperative coronary angiography revealed a 70% stenosis of the left anterior descending coronary artery (LAD), and the right coronary artery (RCA). Transthoracic echocardiography showed a severe aortic valve stenosis with an aortic valve area (AVA) of 0.8–0.9 cm^2^. The intraoperative and the early postoperative course were uneventful, but the patient made vague complaints that could not be sufficiently classified. On postoperative day 10, the patient suddenly developed epigastric pain radiating to the left upper abdomen and to the backside, and features of circulatory shock. Clinical examination was without jugular venous congestion, paradoxical pulse, nor Kussmaul's sign as symptom of a hemodynamically relevant pericardial effusion. Auscultation revealed attenuated bowel sounds in the left upper and lower abdominal quadrants. A broad laboratory profile did not point to any particular diagnoses. Urinary retention or urinary tract infection could be excluded by urinary bladder catheterization.

Crystalloids were administered to maintain cardiac output and organ perfusion, and a transthoracic echocardiographic examination was performed, revealing a small to moderate pericardial effusion. Echo findings suggestive of cardiac tamponade, such as right atrium (RA) collapse during systole, right ventricle (RV) free wall diastolic collapse, irregular movement of the RV free wall, or a dilated inferior vena cava (IVC) were not apparent. The global function of the left ventricle (LV) was preserved with basal septal hypokinesia and the function of the biological aortic valve was inconspicuous. However, the diagnostic validity was limited by a poor acoustic window.

**Fig. 1:**
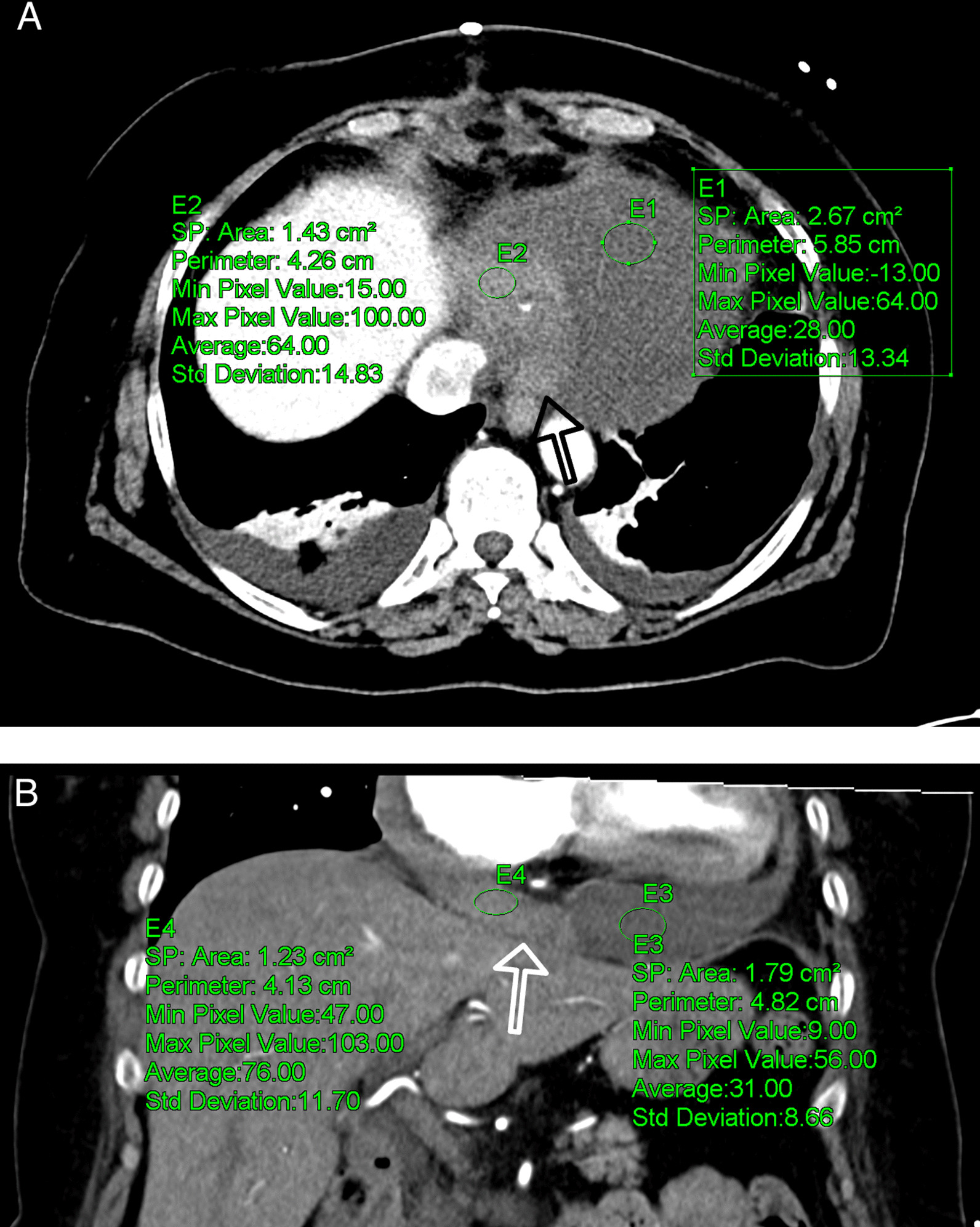
Abdominal CT-scan in axial (**A**) and coronal (**B**) view with contrast medium in portal-venous phase shows increased density (64–76 Hounsfield units) of the fluid in the pericardium (normal fluid 28–31 Hounsfield units), which is compatible with acute hemopericardium ( min.: minimum; max.: maximum; Std. deviation: standard deviation)

**Fig. 2:**
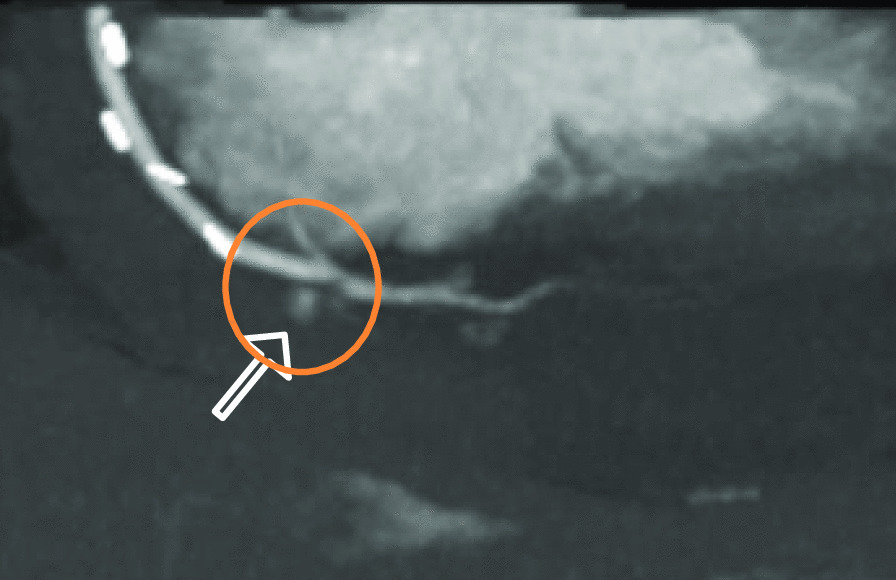
Active extravasation of contrast agent on abdominal CT Scan, arterial phase, paracoronal plane with MIP- reconstruction. (CT: computer tomography; MIP: maximum intensity projection)

**Fig. 3:**
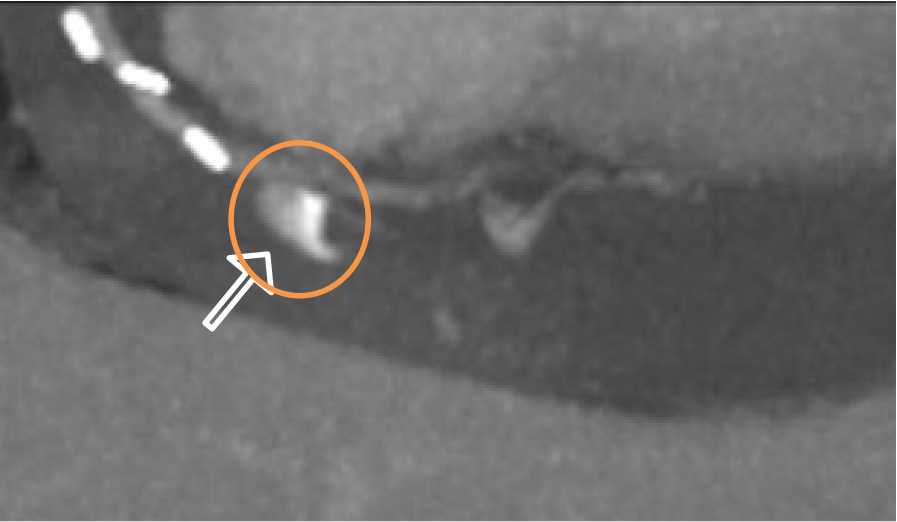
Active extravasation of contrast agent on abdominal CT Scan with blood pooling in portalvenous phase, paracoronal plane with MIP- reconstruction. (CT: computer tomography; MIP: maximum intensity projection)

Because the patient's condition deteriorated and a slight lactate elevation developed, an additional abdominal CT scan (Siemens SOMATOM X.ceed, 128 slices by z-flying focal spot, 0.25 s rotation time, 0.6 mm collimation, Siemens Healthcare GmbH, Forchheim, Germany) was performed to further determine the underlying etiology. The contrast-enhanced computed tomography in arterial and portal-venous phase (100 ml Iomeprol, Imeron^®^ 350, Bracco Imaging SpA, Milan, Italy) was able to rule out abdominal pathology, such as abdominal aortic dissection, mesenteric infarction, abdominal bleeding, acute pancreatitis, nephrolithiasis, or ileus. Incidentally, parts of the heart and pericardium were also visualized in the abdominal scan, revealing a hemopericardium (width 25 mm), resulting from active bleeding distally on the anastomosis of the vein graft to the right coronary artery (Figs. 1, 2, and 3).

While an operating room was immediately provided for emergency reoperation, the patient's condition deteriorated, and she had to be resuscitated by external thoracic compression for 2 min.

Intraoperatively, the source of bleeding was located distally to the anastomosis, suspicious of being an errosive bleed. It was addressed surgically by placing a 6–0 prolene suture. The patient was extubated on the first postoperative day, and the further postoperative course was uneventful.

## Discussion

Pericardial tamponade (PT) occurring early after cardiac surgery is a clinical entity that is challenging, not infrequently misrecognized, and often only detected late in its course, as it is distinct from the manifestation of PT associated with other medical pathologies. Because the clinical signs of pericardial tamponade can be unspecific, a high degree of initial suspicion is required to establish the diagnosis. Recognition of this complication often requires the deployment of imaging techniques in addition to clinical examination in order to avoid delays in diagnosis and to initiate any necessary interventions, such as pericardiocentesis or direct cardiac surgical interventions [1].

Because PT still remains underrecognized outside of cardiac surgery, we provide below a brief overview of how knowledge of this state has developed throughout history. The first description of the pericardium as a cardiac structure can be traced back to the ancient Greeks, where Hippocrates of Kos (circa 460 BC–370 BC), one of the most outstanding personalities in the history of medicine, described it as “a smooth mantle surrounding the heart and containing a small amount of fluid resembling urine [2, 3]”. The Greek physician and anatomist Galenos of Pergamum, also known as Galenus (b. c. 129 in Pergamum, † c. 199 AD in Rome), brought up the pericardium again when he noticed that gladiators had heart injuries, most of which were lethal. He also documented a pericardial effusion of a monkey he had dissected [4, 5]. In the twelfth century, the most well-regarded physician of his era, the Arab physician, surgeon, and poet Ibn Zuhr (1094 in Seville, † 1162 in Seville), traditionally known by his latinized name Avenzoar, wrote in Seville about “water that accumulates in the heart pocket” [4]. Perhaps because the ancient Greeks believed that the heart was inviolable and not a subject for disease, no further descriptions of the pericardium emerged for several centuries. Only very sporadic descriptions of pericarditis or pericardial effusions can be found in the historical literature.

The Renaissance, the period of transition from the Middle Ages to modern times, was followed by an era of enlightenment about the pathophysiology and clinical signs of cardiac tamponade. In the sixteenth century, Ambroise Paré (b. c. 1510; † Dec. 20, 1590), a French royal and military surgeon who is considered a pioneer of modern surgery, reported acute traumatic hemopericardium in a man wounded in a duel. At autopsy, he discovered a wound in the heart “so large as would contain one’s finger, and there was much blood that poured forth upon the midriff” [6]. The tamponade effect of pericardial effusion was first observed in 1669 by the Richard Lower, an English physician who heavily influenced the development of medical science by his work on blood transfusion and the function of the cardiopulmonary system, which he described in his book “*Tractatus de Corde”* [3, 7]. The Italian physician and anatomist Giovanni Battista Morgagni, who is considered the founder of modern pathology and who advocated the idea that every disorder of health should be associated with an anatomical change, identified several causes of hemopericardium and hemotamponade, including the puncture of a coronary artery. He also made the important observation that the outcome of cardiac injury may depend on the degree of pericardial filling [2].

Until the development of modern imaging techniques, the diagnosis of pericardial effusion and tamponade was based on clinical diagnosis and several renowned physicians have contributed to the closer characterization of the typical clinical symptoms and signs and almost all important clinicians and pathological anatomists paid attention to pericardial diseases in the first half of the nineteenth century [4]. The Austrian physician, Leopold von Auenbrugger (b. November 19, 1722, Graz; † May 18, 1809, Vienna), who first to introduced percussion in the medical examination in 1761, Joseph Leopold Auenbrugger, also described clinical signs of pericardial effusion (“Auenbrugger’s signs”) [2]. The German internist and gastroenterologist Adolf Kussmaul (* 22.2.1822 Graben near Karlsruhe, † 20.5.1902 Heidelberg) described in 1873 three patients in whom the pulse disappeared completely at the height of inspiration while the heartbeat remained paradoxically palpable, calling this phenomenon "pulsus paradoxus". The term remains a clinical characteristic of cardiac tamponade to this day but is unfortunately used in an misleading manner [8]. Pulsus paradoxus is actually an augmentation of the physiologic drop in systemic arterial blood pressure during inspiration, rather than, as it implies, a decrease when a rise would be normal [3]. The term “cardiac tamponade” was coined in an 1884 treatise by the German surgeon Edmund Rose (October 10, 1836–May 31, 1914). He presented cases of fatal cardiac injury in which patients did not die from hemorrhage or from the extent of the injury itself, but primarily from compression of the heart by a relatively small amount of blood trapped in the pericardial cavity [9]. In 1935, the American cardiac surgeon Claude Schaeffer Beck (November 8, 1894–October 14, 1971) described the combination of hypotension, an increased venous pressure, and a quiet heart classically associated with acute cardiac tamponade. They are collectively called “Beck's triad” [10]. Later studies have demonstrated however that these classic findings are seen in only a minority of patients with cardiac tamponade.

**Fig. 4:**
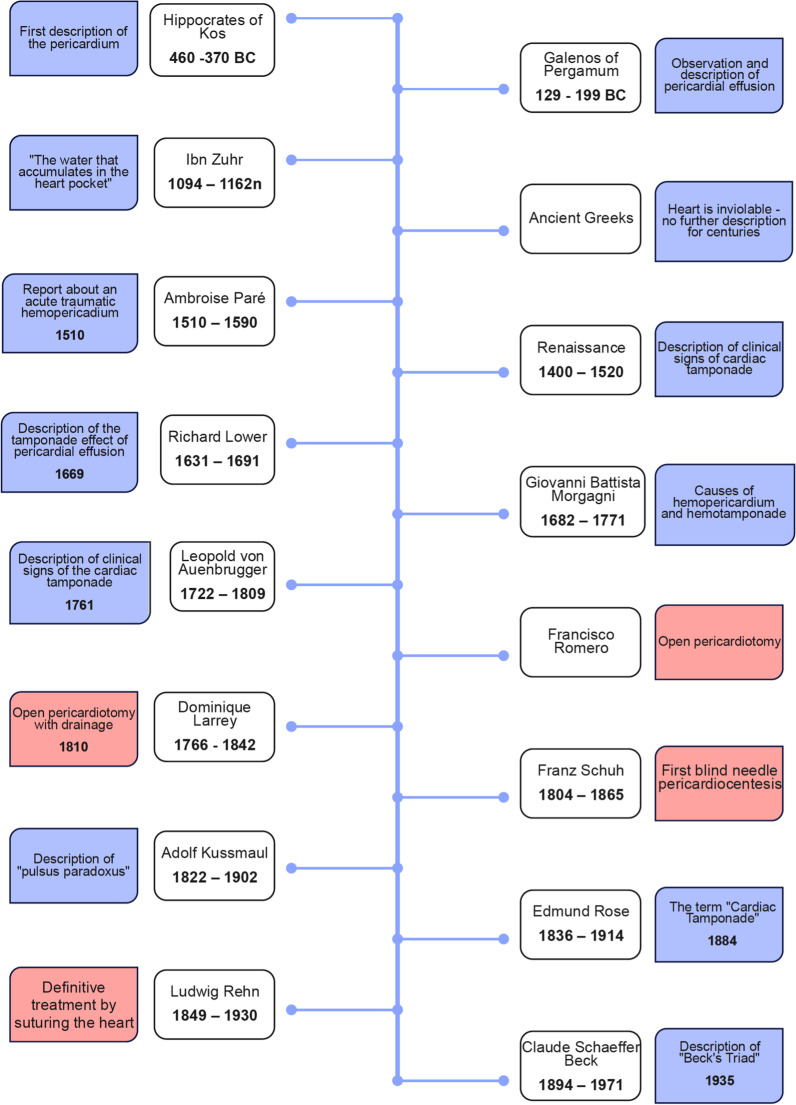
Milestones of the diagnosis and treatment of a cardiac tamponade—blue: historical aspects; red: surgical aspects. (BC: before Christ)

The first treatments of cardiac tamponade date back to the beginning of the nineteenth century. The Catalan Francisco Romero, surgeon at the Royal College of Barcelona and then a military surgeon in Madrid, was the first to successfully perform an open pericardiotomy to treat pericardial effusion in 1801 [11] (Fig. 4). The French surgeon and military physician Dominique Larrey (July 8, 1766–July 25, 1842), chief surgeon to Napoleon Bonaparte, is given credit for a very similar operation in 1810 on a patient whose pericardial cavity had filled with blood after a penetrating wound to the heart. That patient survived 23 days and died from a suppurative pericarditis. Another drainage performed by Larrey in 1824 resulted in a better outcome [12, 13] (Westaby S, Bosher C [12]. Landmarks in Cardiac Surgery. Oxford: Isis Medical Media). (Shumacker HB Jr. [13]. Evolution of Cardiac Surgery. Bloomington, IN: Indiana University Press). The first blind needle pericardiocentesis was performed in 1840 on a 24-year-old woman by Franz Schuh (17 October 1804–22 December 1865), a Viennese pathologist and physician. The procedure, performed by inserting a trocar through the third and then the fourth intercostal space, achieved considerable international attention. The patient improved immediately but later died of mediastinal neoplasm [14, 15]. The definitive treatment of traumatic cardiac tamponade by suturing the heart was first successfully performed in 1896 by Ludwig Rehn (13. April 1849 in Allendorf, † 29. Mai 1930 in Frankfurt am Main) on a 22-year-old gardener with a thoracic step wound. After opening the chest wall, blood was seen leaking from a pericardial tear and a 1.5 cm wound in the right ventricle that was closed with 3 interrupted silk sutures [16, 17]. This landmark operation marks the beginning of cardiac surgery [18] (Fig. 4).

From the course of an adult patient after elective aortic valve replacement and CABG (LIMA to LAD and a single vein graft to RCA) who unexpectedly decompensated on the 10th postoperative day, the particularities of clinical presentation, diagnosis, and management of this rare complication are discussed.

The presented case demonstrates that a high level of suspicion, immediate diagnostic confirmation, and rapid treatment are required to recognize and successfully treat such an emergency.

It is always important to recognize PT in a timely manner and investigated patients´ complaints, such as respiratory discomfort or exceptional fatigue. In this case, vague complaints in combination with a lack of linguistic abilities were difficult to discern although help of a translator was used. PT results from compression of the heart because of congestion of fluid, pus, blood, gas, or tissue within the pericardial cavity, which can have a variety of causes, including previous cardiac surgery, aortic dissection, catheter ablations of atrial fibrillation, trauma, scarring, neoplastic involvement or inflammation, including even coronavirus disease, of the pericardial space among others [20–23].

In the context of the case described, special consideration must be given to the fact that pericardial effusions are common after cardiac surgery. Their incidence is as high as 85%, depending on the methodology used for its detection. However, only few pericardial effusions will become hemodynamically significant and cause PT. In distinction, the reported incidence of PT is much lower, ranging from 0.1 to 8.8% [24]. PT may occur "early," within the first 24 h, or "late", at least 5–7 days after open heart surgery [24]. "Early" PT is typically attributed to bleeding in the setting of cardiac surgery, or a coagulopathy caused by the heart–lung machine or by using anticoagulants for therapeutic reasons. In contrast, "late" PT is most often multifactorial, e.g., due to mediastinal drainage, postoperative anticoagulation, poor anticoagulation control, or postpericardiotomy syndrome [24–26].

The pathophysiologic mechanism causing tamponade is an increase in intrapericardial pressure sufficient enough to compress the heart. When intrapericardial pressure exceeds end-diastolic ventricular pressure, it results in restricted cardiac inflow, diminished intracardiac volumes, decreased stroke volume, and reduced blood pressure [20, 25, 27, 28]. Substantially reduced stroke volume triggers a cascade of compensatory mechanisms to maintain cardiac output and blood pressure. Stimulation of sympathetic nervous system and catecholamines leads to increased contractility, tachycardia, vasoconstriction and clinically to a typical pattern of cardiogenic shock [20]. The typical clinical signs of rapidly developing PT comprises a patient in shock with dyspnea, cough, chest discomfort, cool legs, arms, ears, and nose, as well as peripheral cyanosis jugular venous distention [29–31]. The classic “Beck triad” with diminished heart sounds, hypotension, and jugular vein distention may implicate PT but is only rarely seen [10]. The pulse paradoxus, described as pathognomonic, can be missing or difficult to detect [20, 25, 32]. Neither the historical Kussmaul's sign, a distention of jugular veins on inspiration, nor the Friedreich's sign, an excessive drop in diastolic central venous pressure, are conclusive. The sensitivity of these signs can be low since postoperative PT often results from localized adhesions rather than circumferential pericardial changes or fluid accumulation [19].

As the presented case shows, clinical signs and symptoms have low sensitivity and are often unreliable, and imaging modalities play an important role in assessment PT, particularly echocardiography, computed tomography, magnetic resonance imaging and cardiac catheterization [19]. Echocardiography is usually the method of choice to detect or exclude pericardial effusion. Classic echocardiographic features of tamponade include right atrial systolic collapse, right ventricular (RV) diastolic collapse, a paradoxical motion of the interventricular septum, a swinging heart and an enlarged, non-pulsatile inferior vena cava, and also a reciprocal variation in ventricular size, trans-mitral and trans-tricuspid doppler-flow velocity paradoxus [25, 31, 33–38]. However, these classic echocardiographic hallmarks of PT may be absent, particularly in the postoperative patient. The detection of only a small amount of pericardial fluid or the absence of any classical tamponade signs, does not exclude a hemodynamically relevant PT requiring immediate relief [20, 25].

As the present case also exemplifies, if tamponade is clinically suspected, even if clinical signs and/or echocardiography are inconclusive, further imaging modalities are mandatory, including computed tomography, magnetic resonance imaging, or even cardiac catheterization.

Sophisticated computed tomography and cardiac magnetic resonance imaging may reveal pericardial effusion, distention of the superior and inferior vena cava, reflux of contrast media into the azygos vein and inferior vena cava, deformity and compression of the cardiac chambers and other intrapericardial structures, and angulation or bowing of the interventricular septum [20]. Multi-phasic CT with high temporal and spatial resolution as well as optimal contrast bolus timing is necessary to prove active extravasation of contrast medium and corresponding blood into the pericardium. Due to the larger field of view compared to echocardiography, additional information is provided, including assessment of the entire chest, abnormalities in the mediastinum, lungs, and adjacent structures [20]. Advanced CT-scanners may also deliver cine CT-images and important information about the function and dynamics of the heart and pericardium [20]. Cardiac magnetic resonance imaging may be useful in particular when regional tamponade is suspected in hemodynamically stable postoperative patients [35, 39–42].

Cardiac catheterization is only rarely used as an initial diagnostic test and can reveal equilibration of average intracardiac diastolic pressures (usually between 10 and 30 mmHg) and the inspiratory increase in right-sided pressures and reduction in left-sided pressures.

Failure to recognize the hemodynamic changes in PT or waiting for complete pathognomonic clinical or echocardiographic findings may delay essential treatment and result in serious morbidity and even death from hemodynamic collapse. Timely decompression of PT by echocardiographic or fluoroscopic guided percutaneous pericardiocentesis or subxiphoid thoracotomy or resternotomy may be life-saving [24, 31, 43, 44].

## Conclusion

**Fig. 5:**
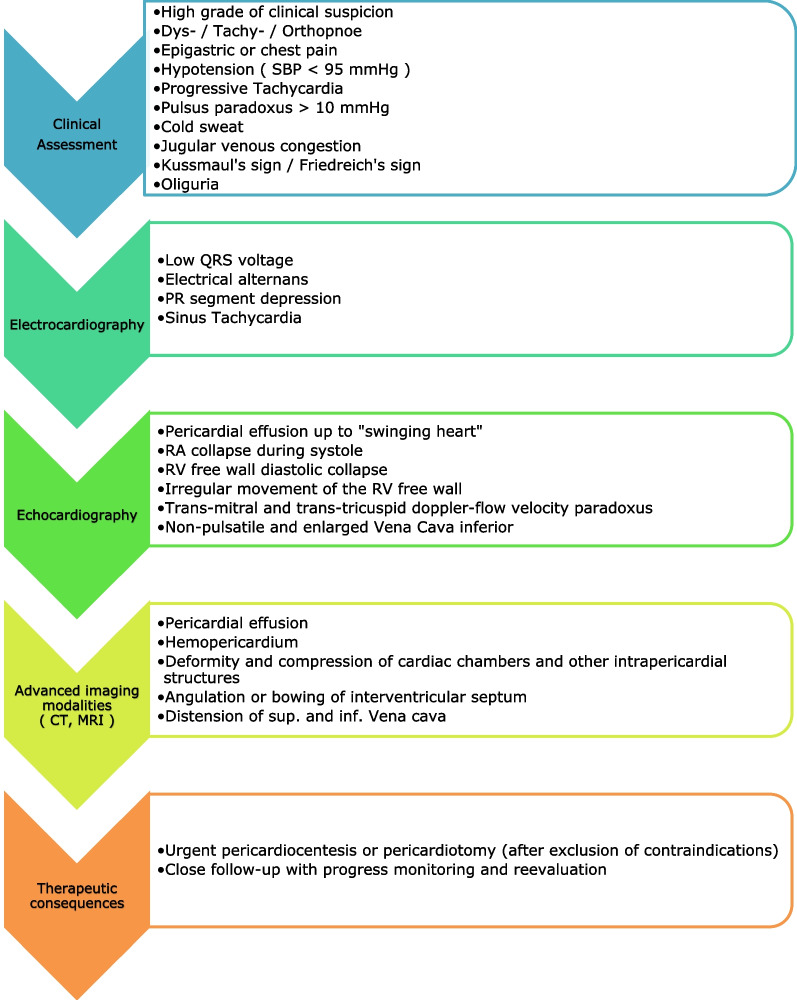
Overview of the symptoms of a cardiac tamponade to diagnosis and treatment of such a condition (SBP: systolic blood pressure; RA: right atrium; RV: right ventricle; CT: computer tomography; MRI: magnetic resonance imaging; sup.: superior; inf.: inferior)

In the first days after cardiac surgery, the occurrence of life-threatening situations such as cardiac tamponade must be expected. Especially if the symptoms are atypical, the entire diagnostic armamentarium must be applied to identify the origin of the complaints, which may be cardiac, or non-cardiac (Fig. 5). As demonstrated by the case presented, in which a CT abdomen with contrast medium was crucial in determining cardiac tamponade as the origin of symptoms, the time window to life-threatening deterioration can be very short. Interdisciplinary collaboration is often imperative to successfully treat patients immediately.

## Data Availability

Data analysis and description of the medical case on the basis of the patient's curve, the findings of the imaging procedures, as well as the operation report. Data sharing is not applicable to this article as no datasets were generated or analysed during the current study.

## Abbreviations

PT: Pericardial tamponade
LIMA: Left internal mammary artery
LAD: Left descending artery
RCA: Right coronary artery
CT: Contrast-enhanced computed tomography
BMI: Body mass index
CABG: Coronary bypass grafting
AVA: Aortic valve area
RA: Right atrium
RV: Right ventricle
IVC: Inferior vena cava
LV: Left ventricle
BC: Before Christ
AD: Anno domini
SBP: Systolic blood pressure
MRI: Magnetic resonance imaging

## References

[1] Stolz L, Valenzuela J, Situ-LaCasse E, Stolz U, Hawbaker N, Thompson M (2017). Clinical and historical features of emergency department patients with pericardial effusions. World J Emerg Med.

[2] Spodick DH (1970). Medical history of the pericardium. Am J Cardiol.

[3] Meyer P, Keller PF, Spodick DH (2008). Empress Sissi and cardiac tamponade: an historical perspective. Am J Cardiol.

[4] Riedel M (2020). Kardiologie: eine Medizin- und Kulturgeschichte.

[5] Acierno LJ (1994). The history of cardiology.

[6] Beck CS (1926). Wounds of the heart: the technic of suture. Arch Surg.

[7] Tubbs RS, Loukas M, Shoja MM, Ardalan MR, Oakes WJ (2008). Richard Lower (1631–1691) and his early contributions to cardiology. Int J Cardiol.

[8] Kussmaul A. Ueber schwielige Mediastino-Pericarditis und den paradoxen Puls: Gedruckt bei Julius Sittenfeld; 1873.

[9] Rose E. Herztamponade: ein Beitrag zur Herzchirurgie: Verlag nicht ermittelbar; 1884.

[10] Sternbach G (1988). Claude Beck: cardiac compression triads. J Emerg Med.

[11] Aris A (1997). Francisco romero, the first heart surgeon. Ann Thorac Surg.

[12] Westaby SB, Cecil. Landmarks in cardiac surgery oxford: Isis Medical Media; 1997.

[13] Shumacker HB. The evolution of cardiac surgery: Indiana University Press; 1992.

[14] Schuh F (1841). Erfahrungen über die Paracentese der Brust und des Herzbeutels. Medzinisches Jahrbuch Kaiserlichen Königlichen Staates Wien.

[15] Kilpatrick ZM, Chapman CB (1965). On pericardiocentesis. Am J Cardiol.

[16] Rehn L (1897). Über penetrierende Herzwunden und Herznaht. Archiv für klinische Chirurgie.

[17] Rehn L (1907). Zur Chirurgie des Herzens und des Herzbeutels. Archiv für klinische Chirurgie.

[18] Blatchford JW (1985). Ludwig Rehn: the first successful cardiorrhaphy. Ann Thorac Surg.

[19] Chuttani K, Tischler MD, Pandian NG, Lee RT, Mohanty PK (1994). Diagnosis of cardiac tamponade after cardiac surgery: relative value of clinical, echocardiographic, and hemodynamic signs. Am Heart J.

[20] Restrepo CS, Lemos DF, Lemos JA, Velasquez E, Diethelm L, Ovella TA (2007). Imaging findings in cardiac tamponade with emphasis on CT. Radiographics.

[21] Gilon D, Mehta RH, Oh JK, Januzzi JL, Bossone E, Cooper JV (2009). Characteristics and in-hospital outcomes of patients with cardiac tamponade complicating type A acute aortic dissection. Am J Cardiol.

[22] Hamaya R, Miyazaki S, Taniguchi H, Kusa S, Nakamura H, Hachiya H (2018). Management of cardiac tamponade in catheter ablation of atrial fibrillation: single-centre 15 year experience on 5222 procedures. Europace.

[23] Diaz-Arocutipa C, Saucedo-Chinchay J, Imazio M (2021). Pericarditis in patients with COVID-19: a systematic review. J Cardiovasc Med (Hagerstown).

[24] Kuvin JT, Harati NA, Pandian NG, Bojar RM, Khabbaz KR (2002). Postoperative cardiac tamponade in the modern surgical era. Ann Thorac Surg.

[25] Price S, Prout J, Jaggar SI, Gibson DG, Pepper JR (2004). 'Tamponade' following cardiac surgery: terminology and echocardiography may both mislead. Eur J Cardiothorac Surg.

[26] Permanyer-Miralda G (2004). Acute pericardial disease: approach to the aetiologic diagnosis. Heart.

[27] Spodick DH (2003). Acute cardiac tamponade. N Engl J Med.

[28] Troughton RW, Asher CR, Klein AL (2004). Pericarditis. The Lancet.

[29] Gandhi S, Schneider A, Mohiuddin S, Han H, Patel AR, Pandian NG (2008). Has the clinical presentation and clinician's index of suspicion of cardiac tamponade changed over the past decade?. Echocardiography.

[30] Reddy PS, Curtiss EI, Uretsky BF (1990). Spectrum of hemodynamic changes in cardiac tamponade. Am J Cardiol.

[31] Adler Y, Charron P, Imazio M, Badano L, Baron-Esquivias G, Bogaert J (2015). 2015 ESC guidelines for the diagnosis and management of pericardial diseases: the task force for the diagnosis and management of pericardial diseases of the european society of cardiology (ESC) endorsed by: the European association for cardio-thoracic surgery (EACTS). Eur Heart J.

[32] Shabetai R, Fowler NO, Fenton JC, Masangkay M (1965). Pulsus paradoxus. J Clin Invest.

[33] Reydel B, Spodick DH (1990). Frequency and significance of chamber collapses during cardiac tamponade. Am Heart J.

[34] Gillam LD, Guyer DE, Gibson TC, King ME, Marshall JE, Weyman AE (1983). Hydrodynamic compression of the right atrium: a new echocardiographic sign of cardiac tamponade. Circulation.

[35] Klein AL, Abbara S, Agler DA, Appleton CP, Asher CR, Hoit B (2013). American society of echocardiography clinical recommendations for multimodality cardiovascular imaging of patients with pericardial disease: endorsed by the society for cardiovascular magnetic resonance and society of cardiovascular computed tomography. J Am Soc Echocardiogr.

[36] Mercé J, Sagristà-Sauleda J, Permanyer-Miralda G, Evangelista A, Soler-Soler J (1999). Correlation between clinical and Doppler echocardiographic findings in patients with moderate and large pericardial effusion: implications for the diagnosis of cardiac tamponade. Am Heart J.

[37] Perez-Casares A, Cesar S, Brunet-Garcia L, Sanchez-de-Toledo J (2017). Echocardiographic evaluation of pericardial effusion and cardiac tamponade. Front Pediatr.

[38] Alerhand S, Carter JM (2019). What echocardiographic findings suggest a pericardial effusion is causing tamponade?. Am J Emerg Med.

[39] Kramer CM, Barkhausen J, Flamm SD, Kim RJ, Nagel E, Society for Cardiovascular Magnetic Resonance Board of Trustees Task Force on Standardized P. Standardized cardiovascular magnetic resonance (CMR) protocols 2013 update. J Cardiovasc Magn Reson. 2013;15:91 10.1186/1532-429X-10-35 PMC2467420 18605997

[40] American College of Cardiology Foundation Task Force on Expert Consensus D, Hundley WG, Bluemke DA, Finn JP, Flamm SD, Fogel MA, et al. ACCF/ACR/AHA/NASCI/SCMR 2010 expert consensus document on cardiovascular magnetic resonance: a report of the American college of cardiology foundation task force on expert consensus documents. Circulation. 2010;121(22):2462–508. 10.1161/CIR.0b013e3181d44a8f PMC3034132 20479157

[41] Maggiolini S, De Carlini CC, Ferri LA, Colombo GI, Gentile G, Meles E (2016). The role of early contrast-enhanced chest computed tomography in the aetiological diagnosis of patients presenting with cardiac tamponade or large pericardial effusion. Eur Heart J Cardiovasc Imaging.

[42] Kamada K, Wakabayashi N, Ise H, Nakanishi S, Ishikawa N, Kamiya H (2020). Routine postoperative computed tomography is superior to cardiac ultrasonography for predicting delayed cardiac tamponade. Int J Cardiovasc Imaging.

[43] Uramoto H, Hanagiri T (2010). Video-assisted thoracoscopic pericardiectomy for malignant pericardial effusion. Anticancer Res.

[44] Gumrukcuoglu HA, Odabasi D, Akdag S, Ekim H (2011). Management of cardiac tamponade: a comperative study between echo-guided pericardiocentesis and surgery—a report of 100 patients. Cardiol Res Pract.

